# Supplementary material for: Potential Anticancer Activity of Pomegranate (Punica granatum L.) Fruits of Different Color: In Vitro and In Silico Evidence
Source: Biomolecules. 2022 Nov 7;12(11):1649. doi: 10.3390/biom12111649 (PMC9687934; doi:10.3390/biom12111649)
Supplement: Supplementary file 1 [file biomolecules-12-01649-s001.zip › biomolecules-1983137-supplementary.pdf]

Table S1a. UHPLC-MS^2 identification of phenolic acids and flavonoids in pomegranate arils of different color[1.]

Compound	*rt*	$[M]_{m/z}$	Ion fragments	Red	Pink	White
			Phenolic acids			
Ellagic acid	5.71	301.999	254.993, 283.996	0.098 ± 0.003^b	0.160 ± 0.006^a	0.095 ± 0.002^b
Ellagic acid glucoside	4.80	463.052	300.998, 299.991, 271.9951	0.538 ± 0.007^c	0.985 ± 0.113^a	0.574 ± 0.014^b
Ellagic acid-4-O-xylopiranoside	5.54	433.042	299.991, 300.999	0.104 ± 0.003^b	0.160 ± 0.019^a	0.103 ± 0.001^b
Gallic acid	1.08	169.014	107.014, 109.029, 125.024	0.011 ± 0.000^c	0.015 ± 0.001^b	0.020 ± 0.002^a
Galloyl 6-O-glucoside	0.84	331.067	169.014, 211.025, 125.024	1.431 ± 0.044^b	2.603 ± 0.446^a	2.771 ± 0.215^a
1,3,6-Trigallaoyl-glucoside	4.13	635.089	313.057, 463.051	0.126 ± 0.006^b	0.159 ± 0.018^a	0.044 ± 0.001^c
Vanillic acid	4.37	167.034	152.011, 108.022, 123.045	1.144 ± 0.014^a	0.029 ± 0.002^b	0.016 ± 0.003^b
			Flavonoids			
Astragalin	4.21	447.093	285.039, 284.032, 255.029, 227.034	0.023 ± 0.000^c	0.082 ± 0.008^a	0.059 ± 0.008^b
Corilagin	1.06	633.073	300.998, 275.019, 463.006	0.053 ± 0.001^b	0.158 ± 0.030^a	0.074 ± 0.002^b
D-(+)-Catechin	3.93	289.071	245.082, 203.071, 109.029, 221.082	0.132 ± 0.002^c	0.144 ± 0.005^b	0.382 ± 0.005^a
(-)-Epicatechin	477	289.071	245.082, 203.070, 109.029	0.045 ± 0.002^a	0.015 ± 0.002^c	0.029 ± 0.002^b
(-)-Epigallocatechin gallate	4.83	457.077	169.014, 125.024	0.005 ± 0.000^a	0.002 ± 0.001^b	0.003 ± 0.000^b
(±)-Naringenin	8.80	271.061	151.004, 119.050, 177.020	0.008 ± 0.001^a	0.001 ± 0.000^c	0.004 ± 0.001^b

Phlorizin	6.92	435.130	167.034	0.117 ± 0.005^{c}	0.162 ± 0.009^{b}	0.201 ± 0.001^{a}
Phellatin	6.68	533.166	515.155, 371.113	0.302 ± 0.012^{c}	1.505 ± 0.095^{a}	1.195 ± 0.068^{b}
Quercitrin	4.20	447.093	243.029, 245.046, 285.040	1.144 ± 0.014^{a}	0.029 ± 0.002^{b}	0.016 ± 0.003^{b}
Quercetin	7.96	301.035	151.004, 178.999, 107.014, 121.030	0.004± 0.000^{ab}	0.001 ± 0.000^{b}	0.004 ± 0.002^{a}

[1]Results are expressed as mean (relative abundance %) ± SD. Different superscript letters within a same row means statistically differences ($p<0.05$).

Table S1b. UHPLC-MS^2 identification of organic acids and anthocyanins in pomegranate arils of different color[1].

**Compound**	***rt***	**$[M]_{m/z}$**	**Ion fragments**	**Red**	**Pink**	**White**
			Organic acids			
Citric acid	0.74	191.020	111.009, 173.009	3.806 ± 0.031^{a}	1.565 ± 0.049^{c}	1.830 ± 0.053^{b}
Malic acid	0.67	133.014	115.004, 71.014, 72.993	0.210 ± 0.003^{b}	0.122 ± 0.012^{c}	0.239 ± 0.008^{a}
			Anthocyanins			
Cyanidin 3,5-O-diglucoside	3.06	611.161	287.055, 449.107	1.507 ± 0.005^{a}	0.053 ± 0.003^{b}	0.013 ± 0.001^{c}
Delphinidin 3-O-glucoside	3.76	465.102	303.050	0.246 ± 0.004^{a}	0.012 ± 0.001^{b}	0.004 ± 0.000^{c}
Delphinidin 3,5-O-diglucoside	1.55	627.156	303.050, 465.102	0.608 ± 0.000^{a}	0.068 ± 0.003^{b}	0.023 ± 0.000^{c}
Pelargonidin 3,5-O-diglucoside	3.36	595.166	271.060, 433.113	0.104 ± 0.000^{a}	0.004 ± 0.000^{b}	0.005 ± 0.000^{b}

[1]Results are expressed as mean (relative abundance %) ± SD. Different superscript letters within a same row means statistically differences ($p<0.05$).

Table S2. Chemoinformatics of pomegranate (PMG) fruit (aryl) phytochemicals[1,2.]

	GI Permeants				**GI Non-permeants**											
	**1**	**4**	**7**	**8**	2	3	5	6	9	10	11	12	13	14	15	16
Molecular weight (g/mol)	302.2	170.1	168.2	290.3	464.3	434.3	332.3	636.5	436.4	534.5	448.4	192.1	611.5	465.4	627.5	595.5
Rotable bonds (#)	0	1	2	1	3	2	8	10	7	7	3	5	7	4	7	7
H-bond aceptor (#)	8	5	4	6	13	12	10	18	10	12	11	7	16	12	17	15
H-bond donor (#)	4	4	2	5	7	6	7	11	7	8	7	4	11	9	12	10
TPSA (Å^2)	141.3	98.0	66.8	110.4	220.5	200.3	185.0	310.7	177.1	210.5	190.3	132.1	272.6	213.7	292.8	252.4
XLogP3	1.10	0.70	1.43	0.36	-0.70	-0.63	-2.26	0.36	0.54	1.27	0.86	-1.72	-2.84	-0.22	-3.19	-2.48
GI/BBB permeant	↑/--	↑/--	↑/--	↑/--	↓/--	↓/--	↓/--	↓/--	↓/--	↓/--	↓/--	↓/--	↓/--	↓/--	↓/--	↓/--
P-gp substrate	--	--	--	Yes	--	--	--	Yes	Yes	Yes	--	--	Yes	--	--	--
CyP inhibitor	1A2	3A4	--	--	--	--	--	--	--	--	--	--	--	--	--	--
LR5V	--	--	--	--	2	2	1	3	1	3	2	--	3	2	3	3
Bioavailability score	0.55	0.56	0.85	0.55	0.17	0.17	0.55	0.17	0.55	0.17	0.17	0.56	0.17	0.17	0.17	0.17
PAINS (alert)	CTA	CTA	--	--	CTA	CTA	CTA	CTA	--	--	CTA	--	CTA	CTA	CTA	--
Leadlikeness	Yes	--	--	Yes	--	--	--	--	--	--	--	--	--	--	--	--
Synthethic accesibility	3.17	1.22	1.42	3.50	5.21	4.94	3.79	5.32	4.93	5.86	5.28	2.18	6.55	5.3	6.59	6.52
GPCR ligand	--	--	--	Yes	--	--	--	--	--	--	--	--	--	--	--	--
Nuclear receptor ligand	--	--	--	Yes	--	--	--	--	Yes	Yes	--	--	--	--	--	--
Protease/enzyme inhibitor	--	--	--	Yes	Yes	Yes	--	--	Yes	Yes	Yes	Yes	--	Yes	--	--

Compounds: Ellagic acid (1), ellagic acid glucoside (2), ellagic acid-4-O-xylopiranoside (3), gallic acid (4), galloyl-6-O-glucoside (5), 1,3,6-tri-O-galloyl-D-glucose (6), vanilic acid (7), D-(+)-catechin (8), phlorizin (9), phellatin (10), quercitrin (11), citric acid (12), cyanidin-3,5-O-glucoside (13), delphinidin-3-O-glucoside (14), delphinidin-3,5-O-diglucoside (15), pelargonidin-3,5-O-diglucoside (16); Catechol-A (CTA), Cytochrome P450 (CyP), gastrointestinal/blood brain barrier (GI/BBB), G-protein coupled receptors (GPCR), Lipinski´s rule of five violations (LR5V), Pan assay interference structures (PAINS), P-glycoprotein (P-gp), high/low (↑/↓), no (--). Generated in SwissADME predictor server (**http://www.swissadme.ch/index.php**) [23].

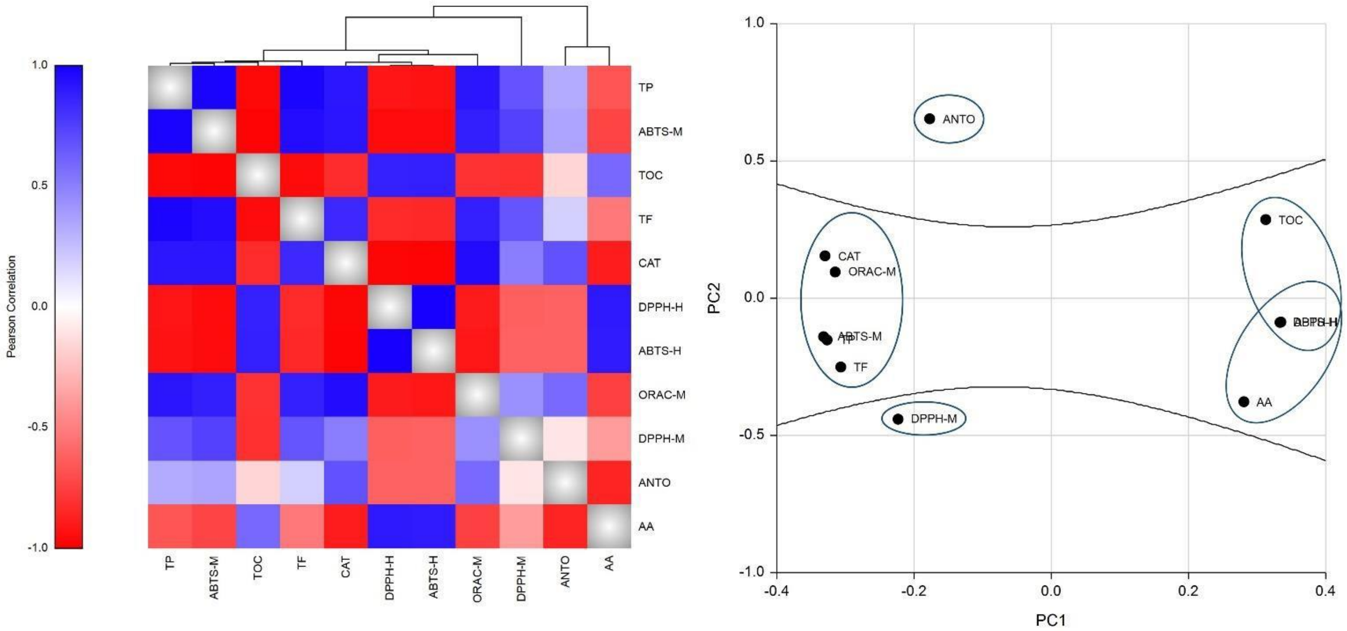

Figure S1. Pearson product-moment correlations between antioxidant phytochemicals (TP, TF,ANTO, AA, CAT, TOC) & total capacity (DPPH-M/H, ABTS-M/H, ORAC-M). Anthocyanins (ANTO), ascorbic acid (AA), total carotenoids (CAT), total flavonoids (TF), total tocols (TOC), total phenolic compounds (TP).

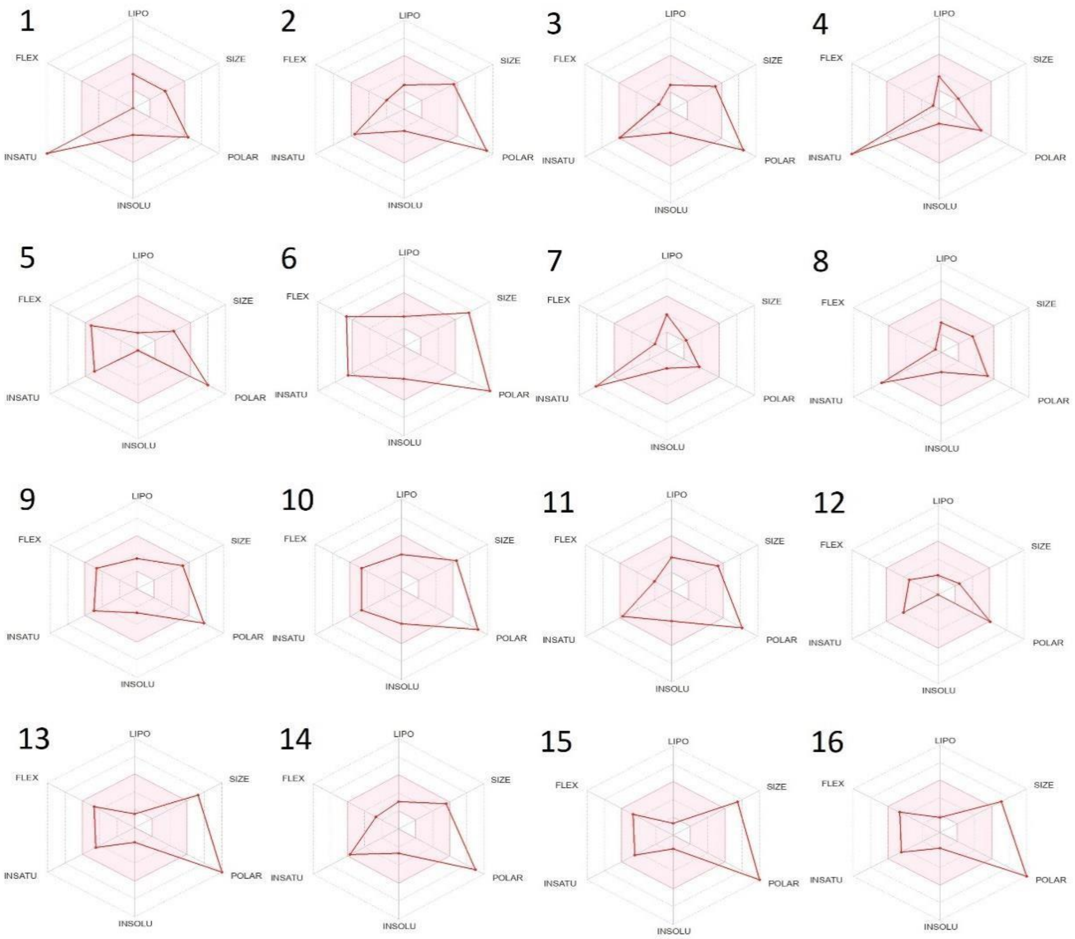

Figure S2. Oral bioavailability (drug-likeness) radar of major phytochemicals in pomegranate fruits of different color. Ellagic acid (1), ellagic acid glucoside (2), ellagic acid-4-O-xylopiranoside (3), gallic acid (4), galloyl-6-O-glucoside (5), 1,3,6-tri-O-galloyl-D-glucose (6), vanilic acid (7),D-(+)-catechin (8), phlorizin (9), phellatin (10), quercitrin (11), citric acid (12), cyanidin-3,5-O-glucoside (13), delphinidin-3-O-glucoside (14), delphinidin-3,5-O-diglucoside (15), pelargonidin-3,5-O-diglucoside (16). The pink area exhibits the zone with the optimal range for a particular property (clockwise; [23]): lipophilicity (LIPO), polarity (POLAR), insolubility (INSOL), insaturations (INSATU), flexibility (FLEX). Gastrointestinal (GI) permeants (Compounds 1,4,7,8). Data obtained with swissADME predictor (http://www.swissadme.ch/index.php).

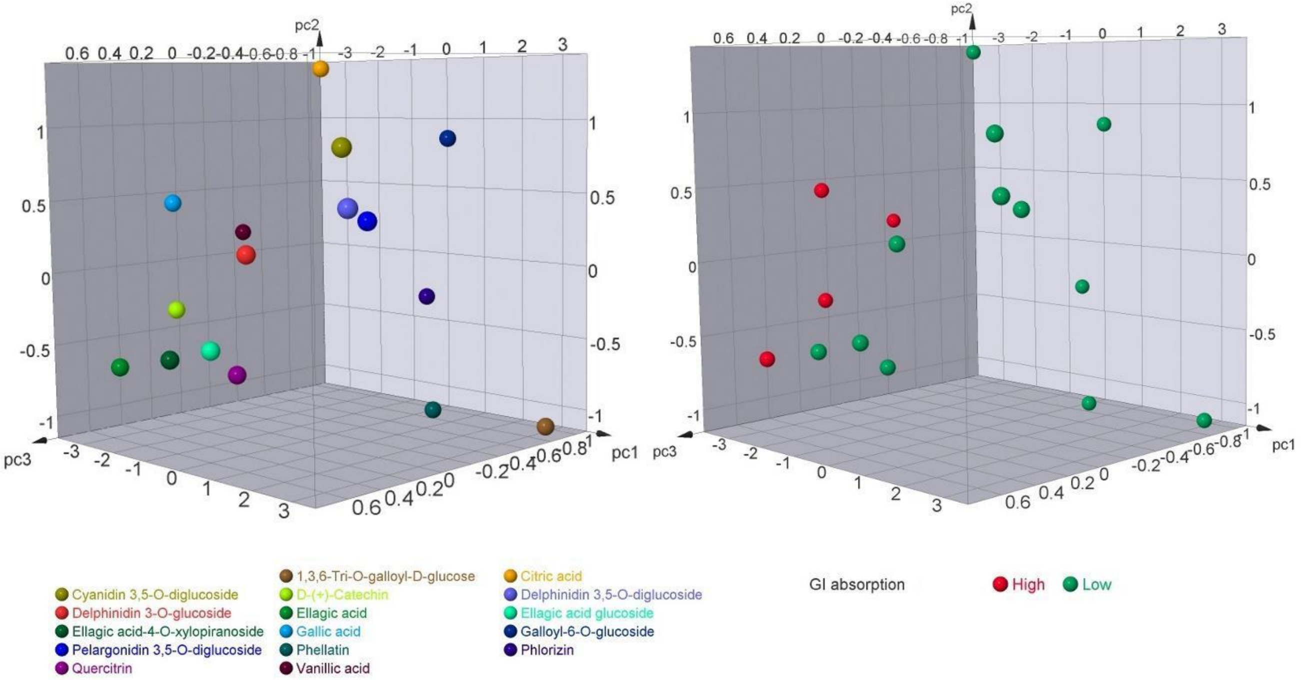

Figure S3a. Chemical space analysis I. Visual representations (principal component analysis; PCA) showing the spatial distribution of major phytochemicals found in pomegranate samples (white-pink-red) were focused on six physicochemical properties (PCP) of pharmaceutical relevance using the SwissADME server [23]: Molecular weight (MW), consensus octanol/water partition coefficient (cLogP), hydrogen bond donor (HBD) and acceptor (HBA), topological polar surface area (TPSA) and rotatable bonds (RB) and predicted GI absorption (right); PCP-based clustering plots were generated with DataWarrior (version 5.2.1) using the t-distributed stochastic neighbor embedding (t-SNE) algorithm [24].

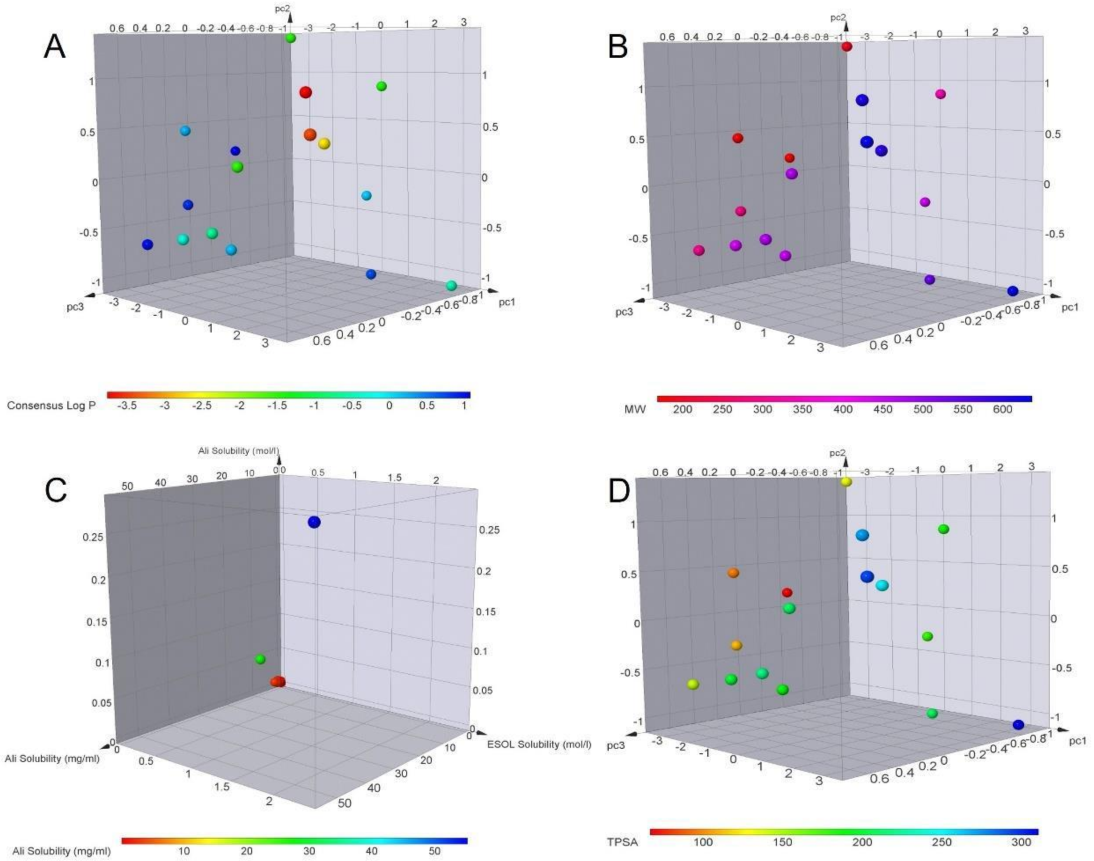

Figure S3b. Chemical space analysis II. Visual representations (principal component analysis; PCA) showing the spatial distribution of major phytochemicals found in pomegranate samples (white-pink-red) According to four physicochemical properties (PCP): consensus octanol/water partition coefficient (cLogP), Molecular weight (MW), solubility and topological polar surface area (TPSA). PCP-based clustering plots were generated with DataWarrior (version 5.2.1) using the t-distributed stochastic neighbor embedding (t-SNE) algorithm[24].
